# Development and Evaluation of a Quadrant Silicon Pad Sensor for the TexAT Active Target Detector

**DOI:** 10.3390/s26103147

**Published:** 2026-05-15

**Authors:** Gyoung Mo Gu, Kyung Yuk Chae, Jong Won Hwang, Kevin Insik Hahn, Jin-A Jeon, Min-Bin Kim, Sunghoon Ahn, Hye Young Lee

**Affiliations:** 1 Department of Physics, Sungkyunkwan University, Suwon 16419, Republic of Korea; kukyoungmo@skku.edu (G.M.G.); kchae@skku.edu (K.Y.C.); 2 Center for Exotic Nuclear Studies, Institute for Basic Science, Daejeon 34126, Republic of Korea; jwhwang@ibs.re.kr (J.W.H.); ihahn@ibs.re.kr (K.I.H.); 3 Center for Underground Physics, Institute for Basic Science, Daejeon 34126, Republic of Korea; jina@ibs.re.kr (J.-A.J.); mbkim@ibs.re.kr (M.-B.K.)

**Keywords:** quadrant silicon sensor, guard-ring design, silicon detector, nuclear physics, TexAT

## Abstract

For low-energy rare-isotope beam experiments, a large-area quadrant silicon pad sensor (5 × 5 cm^2^) has been developed for the TexAT active target system. Unlike finely segmented sensors such as small-scale pad or strip sensors, the operational stability of large-area segmented sensors is critically dependent on the electric field distribution at the device termination; thus, optimizing the guard-ring design is essential to prevent premature breakdown. In this study, we systematically investigated three different guard-ring configurations featuring 6, 9, and 14 rings (denoted as G6, G9, and G14, respectively) through TCAD simulations and experimental measurements. The TCAD results demonstrated that the G9 design, which utilizes a graded-spacing strategy, is more effective in mitigating the maximum electric-field concentration at the sensor edge than designs that simply feature a higher number of rings (G14). Accordingly, the G9-based quadrant sensor was fabricated, and its performance was validated through electrical performance evaluations and radioactive source tests, confirming a low leakage current of several tens of nA and an energy resolution of approximately 31 keV (FWHM) (for 3.18 MeV α-particles from ^148^Gd). Furthermore, beam tests performed at the RAON facility verified the operational reliability of the sensor in a practical in-beam environment. In conclusion, these results provide essential design criteria for large-area silicon detectors in rare-isotope beam experiments, and the developed detectors will be equipped to the TexAT array to enhance the precision of nuclear physics measurements.

## 1. Introduction

In nuclear physics experiments utilizing rare isotope beams, precise measurements of the energy and position of charged particles are essential for reconstructing events of interest. In particular, although large-area silicon detectors are required to ensure wide acceptance, conventional pixel or strip structures face limitations because the number of readout channels and the complexity of the associated electronic circuitry increase significantly as the detector area scales up [1,2,3]. To overcome these constraints, pad-type designs that maximize the single-channel area are considered an alternative.

Previous studies reported the design and validation of large-area pad sensors for electromagnetic calorimeters and high-energy heavy-ion experiments [4,5], as well as the design and electrical characteristics of silicon stripixel sensors with complex large-area structures [6]. Furthermore, cases of a large-area quadrant silicon sensor for charged particle detection have been reported by other researchers [7]. Quadrant silicon pad sensors present a practical alternative by combining energy measurement and essential position-sensing capabilities while maintaining a minimal number of readout channels, effectively reducing the overall complexity of the experimental system configuration.

Building upon these diverse experiences in prior studies, this work describes the design and fabrication of a large-area quadrant silicon pad sensor (5 × 5 cm^2^) specifically optimized for the Texas Active Target (TexAT) system [8] at the Texas A&M University Cyclotron Institute.

The main objectives of this development were to ensure stable leakage-current behavior under high-voltage operation and to achieve a uniform energy response across the four quadrants, together with adequate energy resolution for particles produced in nuclear reaction experiments.

However, compared to finely segmented pad or strip detectors, quadrant sensors with large-area segments face greater challenges in maintaining stability, as their performance is heavily dictated by electric field concentrations at the device edge.

For instance, although the pad sensors (6.2 × 6.2 cm^2^) developed in our previous studies had a larger total area than the quadrant sensor, their active regions were finely divided into 16 or 36 small pads. In these structures, lateral electric field relaxation [9,10] is naturally facilitated by the electric field spreading induced through the many gap spaces between pads in the sensor. Consequently, these sensors could maintain electrical stability using only three guard-rings.

In contrast, the quadrant sensor with its large-area segmentation has limited lateral field dispersion, causing the electric field to concentrate abruptly at the device edge. This significantly increases the risk of premature breakdown. Indeed, when our initial prototype—employing the aforementioned guard-ring design—was tested, premature breakdown occurred at approximately 40 V, which is far below the required full depletion voltage of 150 V.

Therefore, an optimized termination design is essential to ensure a stable operating voltage. Against this background, this study derived various termination design options by step-wise adjustments of the number, width, and spacing of guard- rings. Furthermore, we investigated the optimal guard-ring design strategy to maximize electrical stability by analyzing the electric field distribution characteristics for each design through TCAD simulations [11].

Through this optimization process, a quadrant silicon pad sensor was developed that operates stably at high voltages with low leakage current characteristics. Performance verification of the fabricated sensor was conducted through energy response measurements using radioactive sources in a laboratory environment. Subsequently, experimental validation, including beam tests at the RAON facility (Rare Isotope Accelerator complex for ON-line experiments) [12,13], was performed to confirm the operational reliability of the optimized sensor in an actual accelerator environment. Based on these research results, this paper presents design criteria for large-area, low-channel silicon detectors required in active-target-based nuclear physics environments like TexAT and reports their detection performance for both radioactive sources and beam tests.

## 2. Sensor Fabrication and Design Optimization

### 2.1. Fabrication Process

The quadrant silicon sensor developed in this study was fabricated based on a 6-inch n-type ⟨100⟩ silicon wafer with high resistivity (>5 kΩ·cm) and a thickness of 550 μm (Topsil, Batch No. PP8T121-1, Frederikssund, Denmark). The vertical structure of the sensor follows a p^+^/n/n^+^ configuration. The fabrication process involved approximately 50 single-sided processing steps, including four photolithography stages used to form the main patterns.

The active area was formed by implanting p^+^ boron ions (^11^B) into the high-resistivity n-type bulk substrate. During this process, a thin SiO_2_ screening oxide layer was applied to prevent surface damage caused by ion implantation. The final depth of the p^+^ implanted layer, formed through a high-temperature annealing process, is less than 1 μm. This layer plays a crucial role in forming a uniform electric field across the entire area of the sensor and ensuring stable junction characteristics [14].

The backside of the wafer was converted into an n^+^ layer by implanting phosphorus dopants (^31^P) followed by annealing, thereby establishing an ohmic contact with the rear aluminum (Al) electrode. Subsequently, an SiO_2_ insulation layer was formed on the upper surface of the wafer, and contact holes were opened in the active area. A TiW barrier metal was then deposited to prevent junction spiking caused by aluminum diffusion into the silicon.

Finally, aluminum (Al) with a thickness of about 0.5 μm was deposited on both the front and back sides of the wafer. Since this sensor is designed for particle detection, optical transmission is not required. Therefore, a window structure was applied, where the entire active area is covered with Al metal to prevent charge accumulation on the oxide surface and to block light-induced noise.

### 2.2. Quadrant Sensor Design and Guard-Ring Layout

In terms of design, the quadrant sensor features a total active area of 5 × 5 cm^2^ divided into four segments, each with a size of 2.5 × 2.5 cm^2^. In a sensor structure where the area of individual segments is relatively large, the control of the electric field at the sensor edge determines the overall electrical stability. Therefore, a precise design of the termination structure at the outer regions plays a key role in ensuring high breakdown voltage characteristics and the reliability of the device.

The design strategy was adopted to gradually optimize the number and arrangement of guard-rings aimed to ensure electrical stability at the sensor edge. Through this process, 6-ring (G6), 9-ring (G9), and 14-ring (G14) structures were sequentially developed. These structures differ not only in the number of guard-rings but also in the total width of the termination region. This design evolution resulted from efforts to maximize electric field stability, providing essential baseline data for the subsequent simulation analysis and experimental results.

**Figure 1:**
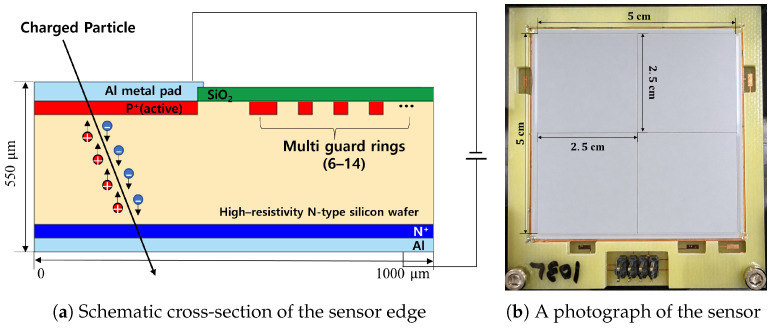
(**a**) Schematic cross-section of the sensor edge with multi-guard-rings, illustrating the physical process of charge carrier generation by an incident particle. (**b**) A photograph of the fabricated quadrant silicon sensor mounted on a PCB.

The schematic configuration and assembly of the developed sensor are detailed in Figure 1. A conceptual cross-section of the sensor edge is illustrated in Figure 1a, while Figure 1b displays the fully assembled quadrant sensor mounted on a dedicated printed circuit board (PCB). This photograph clearly shows the total active area, consisting of four segments, as viewed from above.

**Figure 2:**
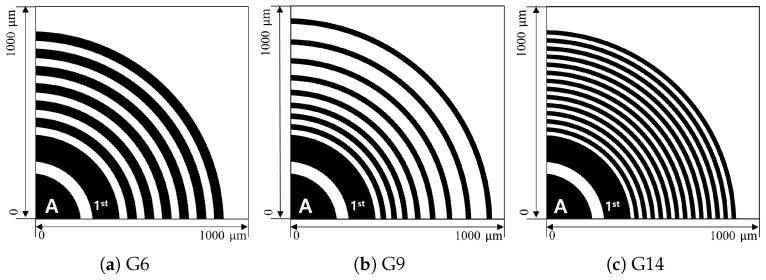
Guard-ring layouts analyzed in this study: (**a**) 6 rings (G6), (**b**) 9 rings (G9), and (**c**) 14 rings (G14) configurations. The region labeled A denotes the active area, and 1st indicates the innermost thick guard-ring used to initiate the termination structure.

Figure 2 presents a comparison of the top views for the three guard-ring designs (G6, G9, and G14). In all three structures, the specifications of the first guard-ring (1st) adjacent to the active area (A) were kept identical. However, the number and arrangement strategy for the remaining guard-ring regions were differentiated.

While the G6 structure is an initial model designed within a relatively limited termination width, the G9 and G14 structures feature expanded termination regions in the horizontal direction and readjusted ring spacing to maximize the efficiency of electric field relaxation at the outer regions. Specifically, the G6 structure features six guard-rings arranged within the termination region. The length scale of this termination region is similar to the sensor thickness (∼550 μm). This design was based on the initial hypothesis that a wider termination region would help relax the electric field toward the sensor edge. Subsequently, the G9 and G14 structures featured guard-rings arranged within expanded termination regions compared to the G6 structure.

The G9 structure consists of nine guard-rings arranged in two regions with different spacing characteristics. Near the active region, the inter-ring spacing was made dense to divide the potential drop more finely and reduce local electric field concentration. Toward the outer edge, the spacing was gradually increased as the potential gradient decreased, allowing the electric field to relax over a wider region. For optimization, the G9 ring width was made narrower than that of G6.

In contrast, the G14 structure maintains the same ring width as G9 but features guard-rings arranged with uniform inter-ring spacing throughout the entire termination region. This design was intended to relax the electric field uniformly and step-by-step across the whole termination area, rather than focusing on a specific region. The impact of this structural evolution on the actual electric field relaxation characteristics is evaluated in detail through the subsequent TCAD simulation analysis.

The main features and operational parameters of the quadrant sensor, including the three guard-ring configurations, are summarized in Table 1 to provide a comprehensive overview of the detector.

### 2.3. TCAD Simulation of Guard-Ring Termination Structures

TCAD simulations were performed to compare and analyze the electric field distribution characteristics according to different guard-ring termination structures [11]. Table 2 shows the key parameters and physical models used in the TCAD simulation. The simulation model incorporated ion implantation, oxidation, and other process conditions consistent with the actual fabrication process. A reverse bias was applied to the front active electrode, while the back side of the sensor was maintained at ground. All guard-rings were set to floating, with no bias applied.

**Figure 3:**
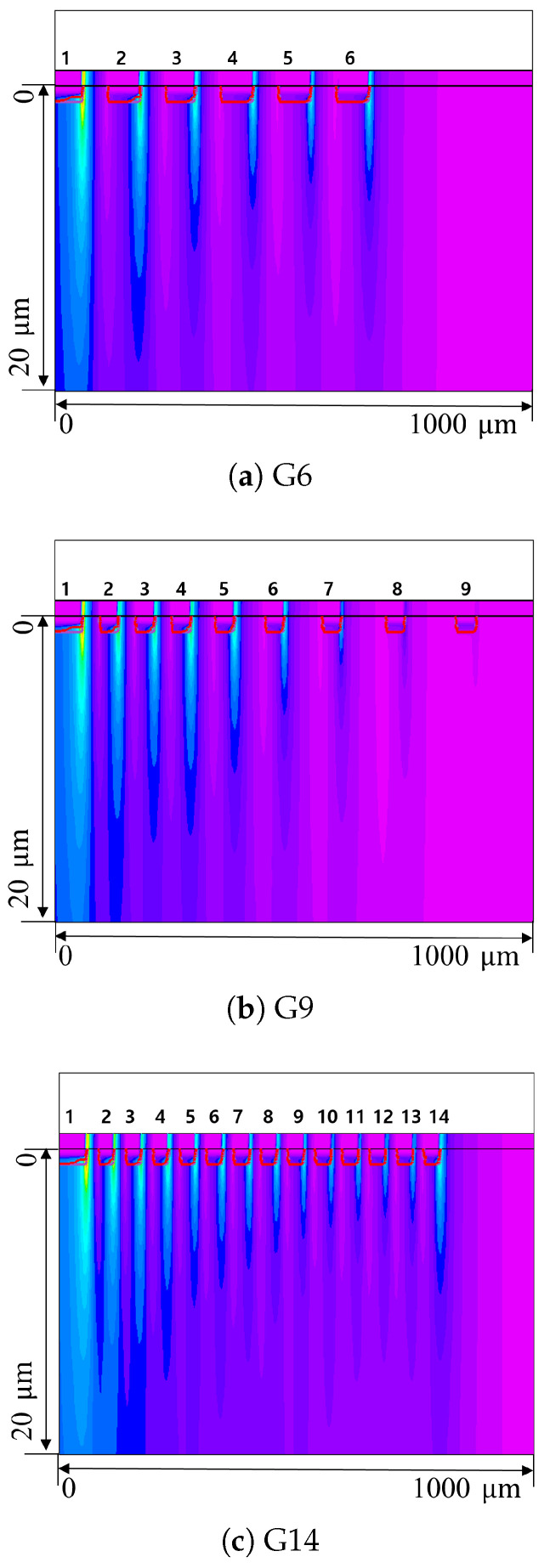
Two-dimensional electric-field distributions in the vertical cross-section for (**a**) G6, (**b**) G9, and (**c**) G14 structures under identical bias conditions. The horizontal and vertical axes represent the lateral distance (1000 μm) from the active region and the sensor depth (20 μm), respectively. All panels share the same spatial and color scales for direct comparison. All panels share the same spatial and color scales for direct comparison. The numbers above the guard-rings indicate the ring index, and the color contours represent the electric-field intensity.

Figure 3 shows the vertical 2D electric field distribution in the sensor termination region. In this figure, the x-axis represents the electric field distribution along the lateral direction of the sensor, while the y-axis represents the distribution along the sensor depth. All three structures exhibit similar electric field distributions near the active area; however, as they approach the outer part of the termination region, the electric field relaxation patterns become distinctly different according to the design strategy of each structure.

**Figure 4:**
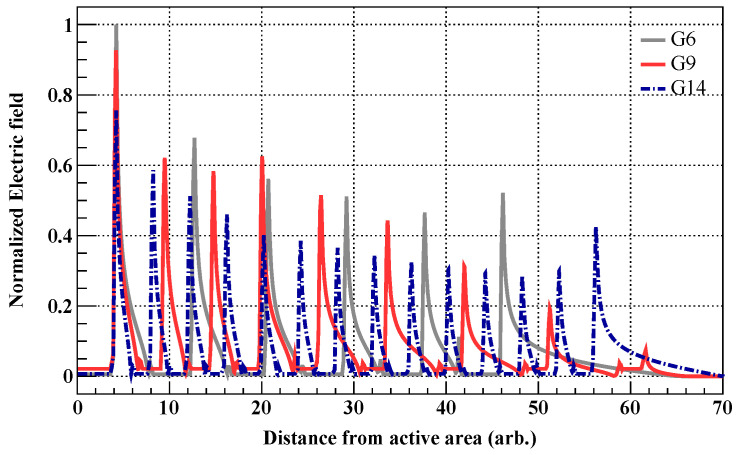
Comparison of 1D electric-field profiles for the three guard-ring designs. The magnitude of the electric field was extracted along the lateral direction at a specific junction depth from the 2D distributions shown in Figure 3.

For a quantitative comparison of the electric-field characteristics of each structure, Figure 4 presents 1D profiles of the electric field magnitude extracted along the lateral direction at a specific junction depth from Figure 3. In this plot, the x-axis represents the lateral distance from the first guard-ring to the end of the termination region in arbitrary units (arb.). The y-axis is normalized to the maximum peak value, allowing for a direct comparison of the electric field-relaxation behavior among the three designs.

As shown in Figure 4, all three designs exhibit similar maximum electric field intensities near the first guard-ring. This result reflects the first guard-ring design, which was applied identically to all structures. However, in the subsequent regions (toward the outer termination), the electric field relaxation characteristics differ distinctly depending on the guard-ring density and inter-ring spacing settings of each design.

In the G6 structure, the electric field remained at a higher level throughout the entire termination region compared to other designs, and a phenomenon in which the electric field increased again at the outermost guard-ring was specifically observed. This suggests that the termination structure ended before the electric field could be sufficiently relaxed within the termination region, causing an electric field crowding effect where the residual electric field locally concentrated at the outermost edge. These results confirm that the initial design hypothesis—setting the termination width based on wafer thickness—has limitations in achieving stable electric field relaxation. Consequently, the findings imply that securing a wider area along with a precise guard-ring arrangement strategy is essential to implement an effective termination structure.

The G14 structure forms a step-like distribution in which the electric field decreases stepwise. As shown in Figure 4, G14 maintains a relatively lower electric field intensity throughout the termination region compared to G6. However, despite the expanded termination area and the larger number of guard-rings, the electric field reduction toward the outer direction remains insufficient. Consequently, an electric field crowding effect was observed near the outermost edge, similar to the G6 structure, where the electric field rises again. This shows that design optimization reflecting the electric-field distribution is necessary, rather than simply increasing the number of guard-rings.

In contrast, the G9 structure exhibits optimized electric field distribution characteristics that maximize efficiency through the previously described two-region design strategy, despite utilizing a smaller number of guard-rings within a termination area similar to G14. Examining the specific electric field distribution, a relatively high electric field intensity was observed in the near-active region, where guard-rings are densely arranged. This demonstrates that the electric field in the region experiencing rapid potential changes is finely partitioned, distributing the potential drop more gradually, as intended by the design. Subsequently, in the outer termination region, the electric field decreased significantly as the guard-ring spacing gradually increased, decreasing to nearly zero near the outer edge. Consequently, the electric field crowding effect at the outermost termination, which was observed in the G6 and G14 structures, is successfully suppressed in G9.

These results confirm that simply expanding the termination region or installing a large number of guard-rings is insufficient to ensure electrical stability, as seen in the comparison between G6 and G14. Rather, as demonstrated in the G9 structure, the electric field stability depends decisively on the strategic inter-ring spacing of the guard-rings within the termination region. Based on these results, all three structures (G6, G9, and G14) were fabricated and evaluated to experimentally verify the comparative analysis of their termination characteristics.

However, these TCAD simulation results are based on a 2D slice model (z-axis thickness: 1 μm) of the sensor edge, and thus have inherent limitations in fully capturing 3D geometric complexities such as corner curvature (Figure 2) and the integrated electric field stress across a large-area device (5 × 5 cm^2^). Consequently, the electric field distribution analysis from these simulations was utilized as a qualitative indicator to verify electric field relaxation effects and guard-ring design effectiveness, rather than to provide an exact quantitative prediction of the breakdown voltage.

### 2.4. Electrical Characterization

The electrical stability and operating parameters of the fabricated quadrant sensor were evaluated through both Current–Voltage (IV) and Capacitance–Voltage (CV) characterizations. The IV curves were measured using a Keithley 6487 Picoammeter/Voltage Source (Tektronix, Beaverton, OR, USA), while the CV characteristics were obtained with an HP Agilent 4277A Precision LCR Meter (Hewlett-Packard, Palo Alto, CA, USA) at a frequency of 1 MHz.

During the measurements, the same reverse bias was applied to all four electrodes, and the total leakage current and capacitance of the device were recorded. In particular, for the leakage current measurements, all guard-rings were left floating without any applied potential. This allowed for the observation of how the electric field relaxation effect, achieved through the self-potential distribution of the guard-rings, influences the leakage current and breakdown voltage characteristics.

**Figure 5:**
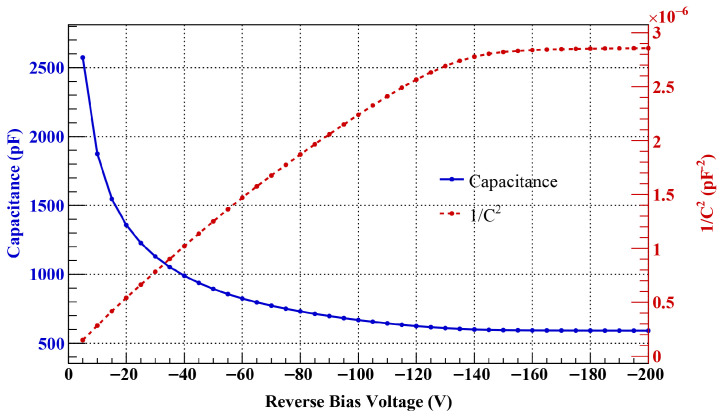
Typical capacitance–voltage (CV) and 1/C^2^ characteristics of the fabricated sensor with a thickness of about 550 μm. The results were obtained from the G9 structure, which features a high bulk resistivity of over 5 kΩ·cm. The 1/C^2^ curve is used to determine the full depletion voltage by identifying the saturation point of the capacitance.

Figure 5 shows the representative capacitance–voltage (CV) characteristics of a sensor fabricated using a substrate with a thickness of about 550 μm and a bulk resistivity of >5 kΩ·cm. The measured full depletion voltage ($V_{fd}$) is about 140–150 V, which is in good agreement with the theoretical value calculated based on the physical specifications of the substrate [9]. In this study, to verify the electrical stability of the sensor, including the operating margin after full depletion, the leakage current (IV) measurement range was extended up to −200 V, which is higher than $V_{fd}$, to evaluate the characteristics of each guard-ring design.

**Figure 6:**
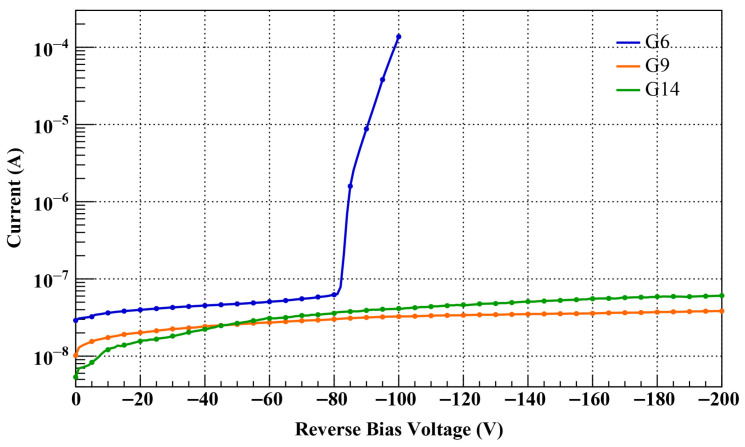
Comparison of current–voltage (IV) characteristics for sensors with G6, G9, and G14 guard-ring configurations. The results show that the premature breakdown observed in the G6 design around −80 V was effectively eliminated in the G9 and G14 designs. The sensors with optimized termination structures maintained stable leakage current at several tens of nA up to −200 V.

The IV characteristics for sensors with the G6, G9, and G14 designs are compared in Figure 6. In the case of the G6 design, a premature breakdown phenomenon was observed, characterized by a rapid increase in leakage current starting near −80 V and reaching several tens of μA at −100 V. This phenomenon suggests that the G6 structure was unable to effectively mitigate the electric field concentration in the termination region well before reaching the full depletion voltage of about −150 V.

In contrast, the sensors employing the G9 and G14 structures exhibited stable electrical characteristics up to the maximum applied voltage of −200 V without breakdown. For both structures, the leakage current remained at the level of several tens of nA over the entire measurement range, with no significant increase observed even after full depletion.

These experimental results are qualitatively consistent with the electric field distribution trends predicted by TCAD simulations. In the simulations, the G6 structure exhibited high electric fields across the entire guard-ring region; in particular, the electric field peak near the outermost guard-ring was locally concentrated without sufficient mitigation. This aligns well with the rapid increase in current observed after −80 V in the measurements.

Similar to the G6, the G14 structure also showed an electric field crowding effect at the last ring in simulations, yet it maintained stable characteristics up to a reverse bias of 200 V. This stability is attributed to the effective distribution of the electric field compared to the G6, resulting from the increased number of guard-rings. Meanwhile, the simulations revealed that the G9 structure suppressed the outermost electric field peak more effectively than the G14 structure. However, this performance difference did not directly translate into a significant difference in leakage current within the actual IV measurement range up to −200 V.

This is because leakage current is likely a complex metric influenced not only by the maximum electric field at the termination but also by bulk generation current, surface states, and the measurement environment. Fundamentally, however, it is interpreted that both structures secured sufficient electric field margins to prevent breakdown within the −200 V range. While the performance difference among the structures may become apparent in a higher voltage environment exceeding −200 V, as shown in the simulation results, both designs demonstrated excellent stability within the operating range of this study.

In conclusion, the IV measurements performed in this study are primarily aimed at preventing premature breakdown and verifying high-voltage operational reliability, rather than quantitatively distinguishing the subtle current differences between guard-ring structures. From this perspective, both the G9 and G14 structures were confirmed to provide sufficient voltage margin and high-voltage stability within the operating range of this study. In particular, the G9 structure simultaneously satisfies the excellent field relaxation characteristics proven in TCAD simulations and the structural efficiency achieved through the optimization of the number of guard-rings. Accordingly, the G9 structure was adopted as the final design in this study, and subsequent performance evaluations were conducted using the sensor manufactured based on it.

## 3. Results and Discussion

This section presents the detection performance of the fabricated quadrant sensor with the optimized G9 structure. Performance was evaluated in two stages. First, measurements using standard α-emitting radioactive sources (^148^Gd and ^241^Am) were performed under controlled laboratory conditions. Second, a beam irradiation experiment was conducted at the RAON facility [12,13] to examine the sensor response under realistic experimental conditions.

In the beam test, the sensor was directly exposed to the incident primary beam. This configuration results in significantly larger energy deposition and potential radiation damage than in typical reaction measurements, where only secondary reaction products are detected. Therefore, the sensor response under direct beam irradiation provides a stringent evaluation of its operational stability and radiation tolerance.

### 3.1. Laboratory Characterization Using Radioactive Sources

The energy response of the fabricated quadrant sensor was evaluated through spectroscopic measurements performed in a laboratory setup using ^241^Am (5.48 MeV, α-particles) and ^148^Gd (3.18 MeV, α-particles) radioactive sources.

**Figure 7:**
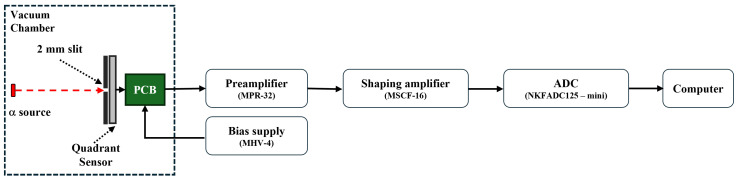
Schematic diagram of the experimental setup for the radioactive source test, showing the signal processing flow from the quadrant sensor to the DAQ system.

Figure 7 shows a schematic diagram of the experimental setup for the radioactive source test. The sensor was installed in a vacuum chamber and operated under a reverse bias of 200 V supplied by a bias power unit (MHV-4), ensuring full depletion of the active volume. When α-particles emitted from the radioactive sources impinge on the sensor, they gradually lose their kinetic energy through energy deposition via Coulomb interactions (dE/dx) [15,16]. This process ionizes silicon atoms within the depletion region, generating electron–hole pairs in proportion to the deposited energy (see Figure 1).

The generated charge carriers are collected by the electrodes under the internal electric field, producing short current pulses that are then sent to a charge-sensitive preamplifier (MPR-32). The preamplifier converts the collected charge into a proportional voltage signal and amplifies it. The signal is subsequently processed by a shaping amplifier (MSCF-16), where it is further amplified and shaped into a Gaussian pulse to optimize the signal-to-noise ratio (SNR).

Finally, the shaped signal is converted into digital data by a data acquisition system (ADC, NKFADC125-mini, Notice Co., Ltd., Anyang, Republic of Korea) and transmitted to a computer, where it is analyzed by constructing energy histograms. To eliminate electrical noise from the system power lines and enhance signal clarity, an independent power supply was provided to the entire system via an Uninterruptible Power Supply (UPS). Furthermore, to compensate for the isotropic emission of the radioactive sources and minimize the energy spread caused by varying incident angles, a collimator with a 2 mm wide slit was used. By restricting the incident area, the intrinsic response characteristics of the quadrant silicon sensor were precisely evaluated.

**Figure 8:**
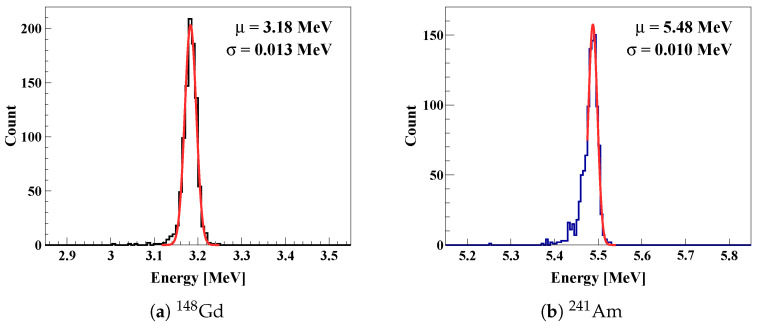
Measured energy spectra of α-particles from (**a**) ^148^Gd and (**b**) ^241^Am sources. In each panel, the black and blue lines represent the raw measured data, while the solid red lines represent Gaussian fits to the primary peaks (3.18 MeV for ^148^Gd and 5.48 MeV for ^241^Am).

Figure 8a shows the representative energy spectrum measured using a ^148^Gd source. The sensor response exhibited a well-defined peak corresponding to the incident α-particles. Gaussian fitting of the peak resulted in a full width at half maximum (FWHM) of about 31 keV, corresponding to an energy resolution of about 1%. This result confirms stable signal formation and efficient charge collection despite the large-area segmented pad geometry.

As shown in Figure 8b, the sensor was also tested with a ^241^Am source to confirm the response to higher particle energies. The extracted energy resolutions were less than 30 keV (FWHM), with variations in peak position and resolution within about 1% among the pads. For ^241^Am, the Gaussian fit was applied specifically to the primary 5.48 MeV peak, excluding the secondary emission at 5.44 MeV to ensure an accurate resolution measurement. This high level of pad-to-pad uniformity demonstrates that the sensor can measure incident particle energy consistently across the entire active area. These results indicate that the fabricated sensor provides a stable and uniform response under operating conditions relevant to active-target detectors such as TexAT. 

### 3.2. Beam Test at the RAON Accelerator Facility

**Figure 9:**
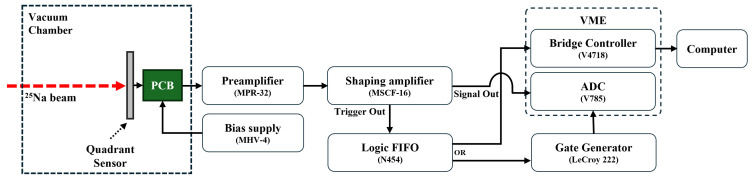
Schematic diagram of the in-beam readout electronics and data acquisition (DAQ) system configuration, illustrating the signal processing flow from the quadrant sensor to the VME-based computer interface.

The sensor was installed at the downstream of the RAON KoBRA (KOrea Broad acceptance Recoil spectrometer and Apparatus) beamline. Responses to the accelerated ^25^Na beam and its associated byproducts were recorded using the in-beam readout configuration shown in Figure 9.

In particular, for direct comparison with laboratory results and control of variables, the front-end electronics configuration, including the preamplifier (MPR-32, Mesytec GmbH, Putzbrunn, Germany) and shaping amplifier (MSCF-16, Mesytec GmbH, Putzbrunn, Germany), was kept identical to maintain consistent experimental conditions. Subsequently, to effectively handle the high counting rates of the accelerator beam and ensure precise timing synchronization with other detectors, a VME-based data acquisition (DAQ) system was established as the back-end electronics.

As illustrated in Figure 9, the signals generated from the incident ^25^Na beam are processed by the shaping amplifier (MSCF-16, Mesytec GmbH, Putzbrunn, Germany), after which the Trigger Out signal is converted into a logic signal for event-by-event synchronization via a Logic FIFO (CAEN N454, CAEN S.p.A., Viareggio, Italy). This signal is split into two paths: one is transmitted to the VME Bridge Controller (CAEN V4718, CAEN S.p.A., Viareggio, Italy) to provide a start point for data readout, and the other is input to the Gate Generator (LeCroy 222, Teledyne LeCroy, Inc., Chestnut Ridge, NY, USA).

In this configuration, the Gate Generator acts as a coincidence or readout window triggered by the Trigger Out signal from the shaping amplifier. Within this gate window, the VME peak-sensing ADC (CAEN V785, CAEN S.p.A., Viareggio, Italy) registers the peak amplitude of the shaped signals (Signal Out), directly recording the energy deposited by the incident particles.

In this study, although the front-end electronics remained identical to the laboratory measurements, systematic effects degraded the energy resolution. Unlike the controlled laboratory environment, the in-beam measurement required mounting multiple distinct detector arrays on a single board inside the vacuum chamber. Because these detectors shared a common bias plane, capacitive coupling and cross-talk between the different detector units significantly increased the system noise. Combined with the complex grounding environment of the beamline, this resulted in an elevated noise floor, degrading the baseline energy resolution to approximately 4% (FWHM) as measured with a ^148^Gd source. This inherent system noise directly contributed to the broadening of the energy peaks observed during the in-beam experiment.

**Figure 10:**
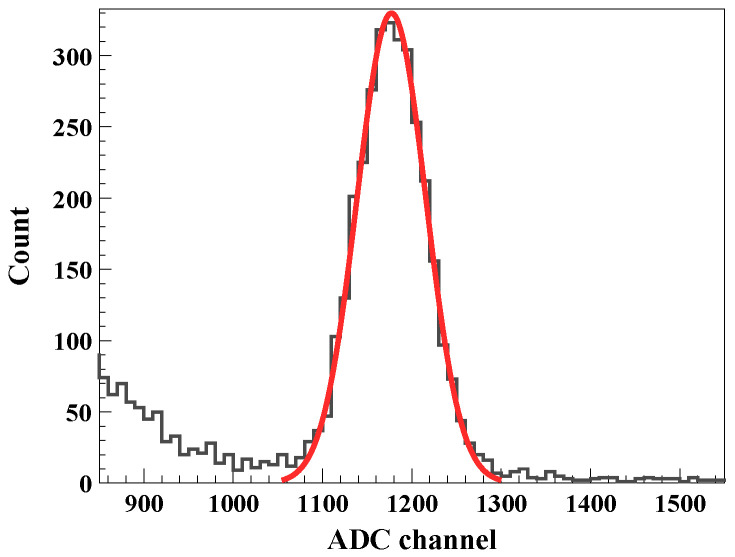
Energy spectrum of ^15^N (37 MeV) and ^25^Na (34 MeV) cocktail beams measured during the RAON beam test. The black line represents the raw measured data, and the solid red line indicates the Gaussian fit to the combined beam peak. The peak shows a widened energy resolution due to system-level noise and energy straggling effects in the experimental facility.

Figure 10 shows the energy spectrum obtained during the beam irradiation. An energy resolution of about 8% was observed in the tens-of-MeV energy region. This relatively poor resolution mainly arises from energy straggling [15,16,17] of the ^25^Na beam passing through the carbon (C) and tantalum (Ta) targets. In addition, the physical overlap between the ^15^N (37 MeV) and ^25^Na (34 MeV) beams further limits the achievable energy separation. The increase in counts observed in the low-energy region near ADC channel 900 is attributed to residual signals from lower-energy species, such as ^40^Ar, whose peak structures were partially suppressed by the hardware threshold. The combined effects of beam–target interactions and the elevated electronic noise floor dominate the observed resolution. A similar resolution was also observed when conventional silicon sensors [18] were operated under the same experimental conditions. This observation indicates that the measured resolution is governed primarily by beam–target interactions and system-level noise rather than by the intrinsic performance of the developed sensor.

Although the energy resolution did not reach the precision of laboratory environments due to the complex systematic factors of the accelerator facility, the key achievement of this study lies in the operational reliability of the sensor demonstrated under realistic experimental conditions. The quadrant silicon sensor maintained stable operation without any measurable increase in leakage current or changes in electrical characteristics, even under direct beam irradiation at an incident rate of approximately $10^{4}$ particles per second (pps). The fact that no performance degradation was observed even after high-dose beam exposure suggests that the sensor possesses sufficient durability and can be operated stably in actual accelerator environments [19]. Future beam tests will be performed with an optimized system incorporating independent bias control for each channel to reduce these systematic effects.

## 4. Conclusions

In this study, a quadrant silicon pad sensor was developed to provide a reduced channel count while maintaining a large active area for the TexAT experiment. Particular attention was given to the optimization of the guard-ring termination structure, since large-area segmented sensors are inherently vulnerable to limited lateral electric-field relaxation and the resulting premature breakdown near the device edge.

For segment structures with dimensions of several centimeters, the electric field tends to concentrate strongly in the termination region. To address this issue, guard-ring configurations with different ring numbers and spacing strategies were systematically investigated through TCAD simulations. The results showed that a structure with dense guard-ring spacing near the active region and gradually increasing spacing toward the sensor edge is the most effective in suppressing electric-field crowding at the outermost termination. These results show that the electrical stability of large-area segmented sensors is governed not simply by the number of guard-rings, but more critically by the inter-ring spacing strategy used to relax the electric field.

IV measurements of the fabricated sensors confirmed that the premature breakdown observed in the initial three-ring design was eliminated in the optimized structure. Stable leakage current at the level of several tens of nA was maintained up to −200 V, which is well above the full depletion voltage. These results verify that the electric-field control strategy established during the design stage plays a decisive role in improving the high-voltage stability of large-area devices. Subsequent measurements with a ^148^Gd source demonstrated an energy resolution of about 1% and channel-to-channel uniformity of about 1%, confirming stable signal formation and uniform sensor response across the full active area.

Beam measurements at the RAON facility further verified sensor operation under realistic accelerator conditions. Although the energy resolution in the beam test was limited by these systematic factors, the quadrant silicon sensor maintained stable operation without any measurable degradation in performance, even under direct beam irradiation at an incident rate of 10^4^ pps. This demonstrates that the sensor possesses sufficient durability to withstand the harsh irradiation conditions of actual accelerator experiments. Future beam measurements will utilize an optimized system with independent bias for each channel to realize the intrinsic high-resolution performance of the sensor. The results of this study not only provide essential foundational data for designing large-area silicon detectors for next-generation rare-isotope experiments but will also be directly implemented in the TexAT detector system to enhance the precision of nuclear physics measurements.

## Figures and Tables

**Table 1:** Summary of the main features and device specifications of the quadrant silicon sensor.

Parameter	Value/Specification
Wafer type	6-inch n-type 〈100〉 silicon
Substrate resistivity	>5 kΩ·cm
Thickness	550 µm
Sensor type	p^+^/n/n^+^
Active area (total)	$5\times 5\;{\mathrm{cm}}^{2}$ (chip dimension: $5.3\times 5.3\;{\mathrm{cm}}^{2}$)
Segmentation	4 quadrants ($2.5\times 2.5\;{\mathrm{cm}}^{2}$ each)
Junction depth	<1 µm (p^+^ implantation)
Metallization	TiW barrier + 0.5 µm Al (window structure)
Full depletion voltage	≈150 V
Guard-ring designs	G6 (6 rings), G9 (9 rings), G14 (14 rings)

**Table 2:** Key parameters and physical models used in the TCAD simulation.

Category	Parameter	Value/Description
Mesh Strategy	Min/Max mesh size	X: 0.1 µm (high-field)/1–2 µm (outside)
		Y: 0.1 µm (high-field)/1–20 µm (bulk)
	Mesh refinement	At junction interfaces and guard-ring edge
Doping Profile	Substrate doping	n-type, $6\times 10^{11}\;{\mathrm{cm}}^{-3}$ (high resistivity)
	P^+^ implantation dose	∼$1\times 10^{15}\;{\mathrm{cm}}^{-2}$
	Junction depth	∼0.8 µm
	Lateral gradient	Lateral diffusion factor (0.8)
Physical Models	Recombination	srh, auger (standard SRH and Auger models)
	Carrier mobility	conmob, fldmob (doping & high-field saturation)
	Impact ionization	selb (Selberherr’s impact ionization model)
	Band gap	bgn (band-gap narrowing effect)
	Tunneling	bbt.std (standard band-to-band tunneling)
Environment	Thermal	Lattice temperature =300 K

## Data Availability

The datasets presented in this article are not readily available because they are part of an ongoing study.
